# The Serum and Antitoxin Treatment of Tetanus

**Published:** 1894-01-20

**Authors:** 


### THE SERUM AND ANTITOXIN TREATMENT OF TETANUS.

A considerable amount of discussion has recently taken place about the treatment of tetanus by injections of the serum of animals which have been rendered immune against this disease. It is well known now that the symptoms of tetanus are due to the action on the body of the toxine produced by the tetanus bacillus. Behring has shown that animals may be rendered immune for tetanus, and his results agree with those of Kittasato. If the serum of an immune animal be injected into other animals it prevents the development of tetanus in them, and even cures this disease after the symptoms are well developed. Behring first showed that to secure immunity a large quantity of serum is necessary; but next he found out that the curative power of the serum increases in direct proportion to the time the animal has been rendered immune for tetanus. In the course of a year Behring has succeeded in obtaining from the horse serum with an immunising power of 1 to 5,000,000, *i.e.*, one gramme of this serum would render immune 5,000 animals, each weighing 1,000 grammes.

Rotter[1] has quite recently related a case treated by Behring's serum. On the sixteenth day after the infliction of the wound 66 grammes were injected, on the seventeenth day 50, on the eighteenth day 45, and on the nineteenth and twentieth days 50. The patient recovered, but it is doubtful whether he was severely ill; anyhow this case shows that the injections do no harm.

Moritz[2] has also recorded three cases treated by Behring's serum, but of these one was chronic and two were very mild. Other observers have used Behring's serum or blood, but many of these cases were unsatisfactory, for in some the serum was very weak, in others the dose was very small, in others the patients were very slightly ill, and in others the treatment was not begun until it was too late. In a few of the cases there was fair evidence that the treatment was beneficial. As an instance we may take one recorded by Barth.[3] A boy, aged eighteen, in whom no wound of any sort was found, was seized with tetanus on January 9th, large doses of chloral and bromide were administered, but he got so much worse that on January 16th Roux[4] injected under the skin of the abdomen three doses of 50 c.c. of serum each, taken from an animal immune for tetanus. Next day a similar injection was given and the patient was better, in the evening another injection was given and the patient was bled. It was found that his blood rendered animals immune against tetanus. On the 18th, as he was better, no injections were made, but they were repeated on the 19th and 21st, from which date the patient gradually got well. It will be noticed that this patient was getting worse day by day at the time the injections were begun.

The cases which have given rise to most criticism are those recorded by Italian observers. Of these there are eleven or twelve. The antitoxin used is that prepared by Tizzoni and Cattani of Bologne. It is the alcoholic extract prepared from the serum of immune animals, and sometimes both the serum and the precipitated antitoxin are used in the same cases. Much doubt has been cast upon these Italian cases by several critics, for many of them were so mild that even the diagnosis seems open to question. The last, that of Gattai,[5] appears to have been the most acute. The antitoxin treatment was begun on the ninth day of the disease, and by the sixteenth day the patient was well. She began to improve the day after the first injection was given. A review of the whole question shows the impartial critic that there is no doubt that animals can be rendered immune by serum containing tetanus antitoxin, but that it is by no means sure that this antitoxin can in any large number of instances save human cases, but that as it does no harm it is worthy of more extended trial. MM. Roux and Vaillard of the Pasteur Institute have kindly offered to supply the serum.

[1] Deut. Med. Wochensch, 1893, p. 152. [2] Münchener Med. Wochensch, 1893, p. 561. [3] Sem. Med., March 8th, 1893. [4] Ann. de l'Inst. de Pasteur, 1893, pp. 102-110. Centralbl. f. Bakt., August 1st, 1893.

